# Analysis of CATMA transcriptome data identifies hundreds of novel functional genes and improves gene models in the Arabidopsis genome

**DOI:** 10.1186/1471-2164-8-401

**Published:** 2007-11-02

**Authors:** Sébastien Aubourg, Marie-Laure Martin-Magniette, Véronique Brunaud, Ludivine Taconnat, Frédérique Bitton, Sandrine Balzergue, Pauline E Jullien, Mathieu Ingouff, Vincent Thareau, Thomas Schiex, Alain Lecharny, Jean-Pierre Renou

**Affiliations:** 1 Unité de Recherche en Génomique Végétale (URGV), UMR INRA 1165-CNRS 8114-UEVE, 2 Rue Gaston Crémieux, 91057 Evry Cedex, France; 2 Unité de Mathématiques et Informatique Appliquées (MIA), UMR AgroParisTech-INRA518, 16 Rue Claude Bernard, 75231 Paris Cedex, France; 3 Chromatin and Reproduction group, Temasek Lifesciences Laboratory, 1 Research Link, 117604 Singapore; 4 Université Paris-Sud, Institut de Biotechnologie des Plantes (IBP), UMR CNRS-UPS, Bâtiment 630, 91405 Orsay Cedex, France; 5 Unité de Biométrie et Intelligence Artificielle (BIA), INRA, Chemin de Borde-Rouge-Auzeville, 31326 Castanet-Tolosan Cedex, France

## Abstract

**Background:**

Since the finishing of the sequencing of the *Arabidopsis thaliana *genome, the Arabidopsis community and the annotator centers have been working on the improvement of gene annotation at the structural and functional levels. In this context, we have used the large CATMA resource on the Arabidopsis transcriptome to search for genes missed by different annotation processes. Probes on the CATMA microarrays are specific gene sequence tags (GSTs) based on the CDS models predicted by the Eugene software. Among the 24 576 CATMA v2 GSTs, 677 are in regions considered as intergenic by the TAIR annotation. We analyzed the cognate transcriptome data in the CATMA resource and carried out data-mining to characterize novel genes and improve gene models.

**Results:**

The statistical analysis of the results of more than 500 hybridized samples distributed among 12 organs provides an experimental validation for 465 novel genes. The hybridization evidence was confirmed by RT-PCR approaches for 88% of the 465 novel genes. Comparisons with the current annotation show that these novel genes often encode small proteins, with an average size of 137 aa. Our approach has also led to the improvement of pre-existing gene models through both the extension of 16 CDS and the identification of 13 gene models erroneously constituted of two merged CDS.

**Conclusion:**

This work is a noticeable step forward in the improvement of the Arabidopsis genome annotation. We increased the number of Arabidopsis validated genes by 465 novel transcribed genes to which we associated several functional annotations such as expression profiles, sequence conservation in plants, cognate transcripts and protein motifs.

## Background

Since the finishing of the whole genome sequencing of the model plant *Arabidopsis thaliana *and its first annotation by the international Arabidopsis community [1], gene prediction results have been regularly updated [2]. Indeed, the MIPS and the TIGR have made available a new annotation release each year taking into account the completion of the genome sequence, the improvement of gene prediction tools and the increasing number of transcript sequences in the database [3]. The latest version is based on recent annotation carried out by TAIR [4]. In addition to this global semi-automatic annotation, different works have also improved Arabidopsis gene detection using orphan ESTs [5,6], comparative genomics [7,8], or combination of data through expertise of gene families [9].

In the framework of the European CATMA project [10,11], a micro-array was produced with 24576 specific gene sequence tags (GSTs). These GSTs were defined from the Arabidopsis genome sequence to be highly specific in order to minimize cross-hybridization [12]. The GST design was based not only on the TIGR annotation, but also on the predictions of protein coding genes obtained with the Eugene v1.0 software [13]. Indeed, by combining different information (transcripts, splicing sites, translation initiation sites, coding potential and protein similarities), Eugene has provided an alternative Arabidopsis genome annotation. By comparing with the TAIR version 6.0 annotation release, the CATMA v2 GSTs tag 21 260 Arabidopsis TAIR genes and 677 regions defined up to now as intergenic. These 677 GSTs, specific to the CATMA resource, are excellent tools to reveal possible under-predicted functional genes in Arabidopsis. Furthermore, several predicted genes are tagged by at least 2 distinct GSTs, most often one overlapping each gene extremity. Previous works on gene annotation pointed out that erroneous gene merging is a usual shortcoming of gene predictors [14,15]. With different GSTs associated with the same genes, we have a powerful way to identify such critical situations.

Available public transcriptome data produced with the CATMA micro-arrays were used to investigate these questions [16]. The dataset of 1044 hybridizations using 522 different samples covers numerous developmental stages, biotic and abiotic stresses and mutants. All the micro-array experiments were performed in our laboratory with a normalized protocol of labeling, hybridization, data normalization and statistical analysis ensuring a perfect homogeneity of the data.

## Results and Discussion

### Selection of candidate GSTs

**Figure 1:**
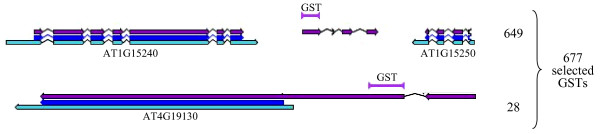
**Selection of GSTs outside TAIR gene models**. The CATMA GSTs are selected in two situations in which they are likely to improve the genome annotation: GSTs designed in a Eugene model between two TAIR genes (649 cases) or GSTs designed in the 5' or 3' CDS extension of a TAIR gene (28 cases). TAIR CDS and mRNA models are represented by dark blue and light blue arrows respectively. Eugene CDS models are represented by purple arrows. Black lines are introns.

Candidate GSTs were extracted from the FLAGdb^++ ^database [17,18]. FLAGdb^++ ^also contains TAIR gene annotations, available transcript sequences and the latest version of the Eugene predictions (v1.59) for the Arabidopsis genome. The gene extremities were extended using overlapping cognate transcript sequences (EST and cDNA). This improved definition of UTRs allowed us to discard GSTs which are outside annotated CDS but which overlap extended transcriptional units. Similarly, GSTs mapped less than 300 bp away from the extremity of a predicted CDS without cognate transcripts were not selected since they could correspond to the unknown UTR region of the corresponding mRNA. The 677 GSTs mapped outside TAIR annotated genes, pseudogenes or known RNA genes (tRNA, snRNA, snoRNA, rRNA and miRNA) were selected as novel candidate genes. Among these 677 candidates, 28 occur in Eugene models, which extend TAIR CDS models. The corresponding expression data provide an improvement of CDS annotation (Figure 1).

### Characterization of novel genes

**Figure 2:**
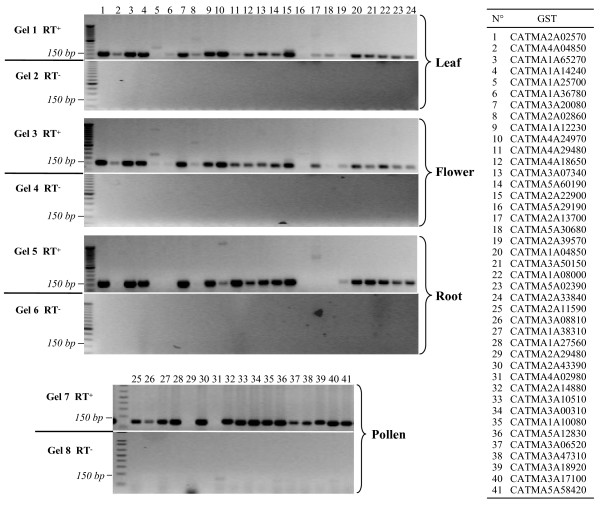
**Validation of novel genes by RT-PCR approach**. PCR were carried out from RT products (RT+: gels 1, 3, 5 and 7) or negative controls (RT-: same reaction but without reverse-transcriptase, gels 2, 4, 6 and 8) from mRNA of leaves, roots, flowers and pollen. Primer pairs 1 to 24 have been used for leaves, roots and flowers, while the 25 to 41 pairs have been used for pollen (for pollen-specific genes only). The table to the right of the figure indicates the correspondence between a primer pair and the corresponding CATMA probe (GST). Primer sequences are given in the additional file 1.

The transcriptome data obtained with the selected GSTs for 522 hybridized samples coming from 40 different experimental projects have been extracted from the CATdb database [16] and analyzed by a dedicated statistical protocol (see the Methods section). Among the 649 candidate GSTs not in extensions of TAIR models, 465, *i.e*. 72%, showed hybridization in at least one sample and probably point out novel genes. To validate the transcriptome results, we performed a RT-PCR for each of the 465 putative novel genes using 4 different mRNA samples from roots, leaves, flowers and pollen. We obtained amplicons for 411 genes (examples in Figure 2) and sequenced all of them. For 410, we obtained a RT-PCR product with a sequence matching the expected target. Thus, for 88% of the putative novel genes, we obtained a proof of transcription by two different experimental approaches.

**Figure 3:**
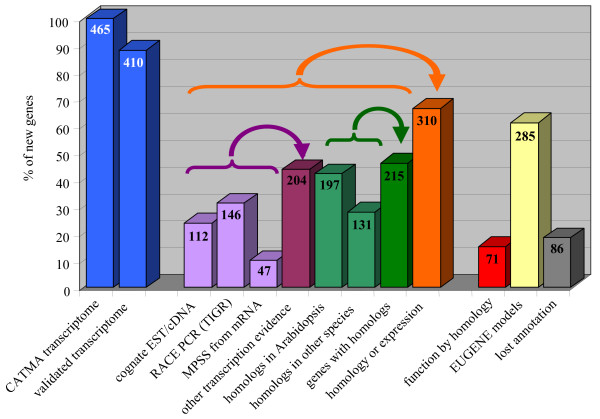
**Structural and functional information about the 465 novel genes detected by CATMA transcriptome data**. The validated transcriptome fraction is the result of our RT-PCR approach. The other evidence of transcription comes from cognate EST/cDNA, RACE PCR from TIGR or MPSS data (purple columns). They are summed up in the "other transcription evidence" class. The fractions of the novel genes sharing similarities with other genes (in Arabidopsis and/or in other species) are indicated in green. The orange column highlights the fraction of novel genes for which there is an indication complementary to CATMA data (homology or transcription) of the gene presence.

To further characterize the newly identified genes, we performed additional data-mining (Figure 3). Other independent evidence of transcription was found for 204 genes (44%) through cognate ESTs or cDNAs, MPSS tags [19] or RT-PCR products recently obtained by TIGR [20]. Indeed, TIGR used RACE-PCR to test 1071 Arabidopsis gene models only predicted by the Twinscan [21] or Eugene [13] programs. The intersection between the 256 novel genes found by the TIGR approach and the 465 novel genes from this work only concerns 146 genes (Figure 3) for which we confirm gene localization and add their conditions of expression in 522 samples.

Sequence comparisons at the protein level and a search for PFAM motifs [22] were applied to each newly identified gene. For 215 genes (46%), significant similarities were detected at least in one other locus in the Arabidopsis genome and/or with proteins from different species, indicating that they belong to known gene families (Figure 3). Nevertheless, inference of function by similarity could be made for only 71 genes (15%) and the remaining 394 genes encode proteins with unknown biochemical function. Surprisingly, 86 genes (18%) were previously annotated by AGI members at the BAC scale (Figure 3) but their model was ignored in the whole genome annotation done later, probably because of poor supporting data.

In 61% of the cases, the latest Eugene v1.59 annotation provided a gene model. In the remaining 39%, we have evidence of the presence of transcriptional units overlapping the GST position but not any additional information on their intron-exon structure. Between the Eugene version used to design CATMA GSTs and the latest Eugene version, the number of false positive predicted genes decreased but some true positive genes were lost.

**Figure 4:**
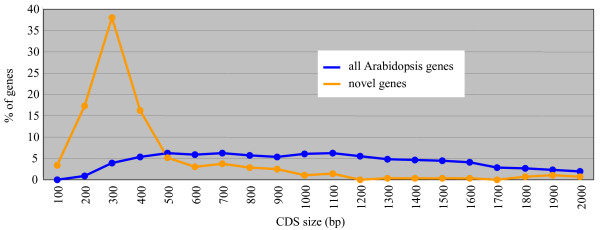
**Size distribution of the novel genes**. Relative distribution of CDS size (in bp) of all Arabidopsis genes (in blue) and of the novel genes (in orange) for which Eugene v1.59 has predicted an intron-exon structure.

Based on the Eugene predicted models, the newly discovered genes are mainly characterized by their short size with a CDS average of 411 bp compared to 1247 bp for the already known Arabidopsis CDS (Figure 4). Consequently, the mean intron number is quite low with 0.67 introns per CDS (191 genes are intron-less) compared to an average of 4.2 for all the annotated CDS. This result could explain why these genes were missed by automatic annotation. Indeed, their coding potential (CDS of unusual length surrounded by larger intergenic regions) may be difficult to detect by a semi-HMM and sequence comparisons are quite likely to generate hits with low scores not or under considered in the gene prediction process. Furthermore, mRNA materials used for EST libraries are usually selected against small size mRNA. Beyond this, our approach has also detected large conserved genes such as CDS of 9 and 11 exons encoding an importin and an ATPase respectively (see Additional File 1).

The topological distribution of the 465 novel genes is quite similar to all the Arabidopsis coding genes. They are evenly distributed in the 5 chromosomes and are rarely present in the peri-centromeric regions or other identified heterochromatic regions.

In 16 additional cases, expression signals associated with candidate GSTs have highlighted an erroneous annotation of the neighbor gene and have led to the improvement of gene models by significant extension of their respective CDS. The extension of these 16 CDS (by one to 4 exons in 3' or 5') is always confirmed by the coherent extension of similarities with homologous proteins (see Additional File 2).

### Expression of novel genes

**Figure 5:**
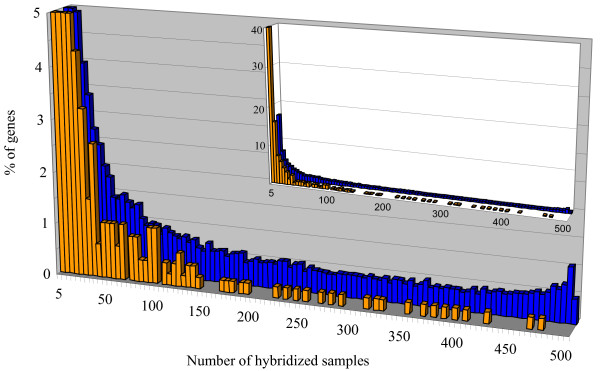
**Expression range of the novel genes**. The genes are distributed according to the number of samples (out of 522) in which they have been found expressed. Values for % of genes are truncated at 5%. Data for all the Arabidopsis genes are represented in blue and those for the 465 novel genes are represented in orange. In the inserted chart % of gene values are not truncated.

**Figure 6:**
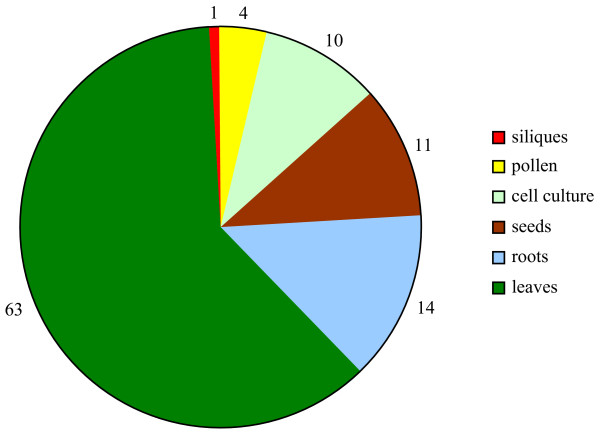
**Distribution of the 103 novel genes expressed in only one organ**. Out of the 465 novel genes, the CATMA transcriptome data have highlighted 103 novel genes found expressed in only one organ.

The comparison of the transcription data obtained from 522 hybridized samples for the 465 novel genes and all the 21 260 Arabidopsis genes tagged by a CATMA v2 GST shows interesting features at the functional level. Most newly identified genes are detected in a limited number of experimental conditions (Figure 5), even if the RT-PCR results may show a basal level of transcripts due to a higher sensitivity. Indeed, 40% of the novel genes have been detected in 1 to 5 mRNA samples while there are only 16% of all the Arabidopsis genes in this category. Furthermore, only 24 genes (5% out of the 465 novel genes) have been detected in more than 30% (150 hybridizations) of the analyzed conditions. This number is very low compared with the 28% of all the Arabidopsis genes that are detected in the same number of conditions. The tail of the distribution in Figure 5 clearly shows that the novel genes identified by this work are never detected in more than 95% of the hybridized samples. Thus, they do not belong to the category of constitutively expressed genes also frequently referred to as housekeeping genes. We have found 103 novel genes (22 %) for which expression is reported in only one organ. Even if we cannot conclude that there is complete organ-specificity from our data, the transcription of these 103 genes is clearly highly preferential in only one organ. Indeed, the observed distribution of the transcription of the 103 genes between the different organs (Figure 6) is not simply explained by the distribution of the 522 hybridization samples among the different organs (P-value = 10^-12^). For instance, we found 63 novel genes expressed in one leaf sample only, which is significantly more than the 38.6 expected (P-value = 0) based on the 136 leaf samples.

**Figure 7:**
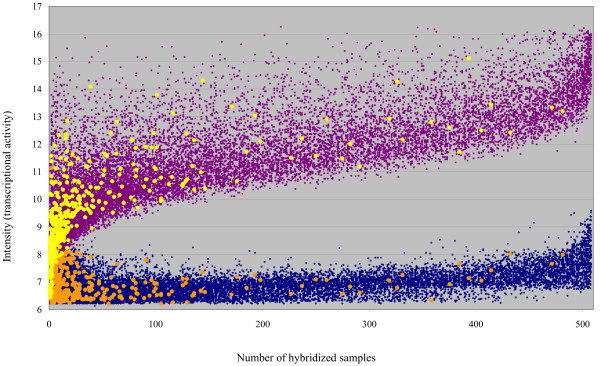
**Expression intensity and expression range of the novel genes**. Each gene is spotted according to the number of samples in which it has been detected and to the hybridization intensity (transcriptional activity): The minimum intensity in any of the hybridizations is in blue and orange for all the Arabidopsis genes and the 465 novel genes respectively. The maximum intensity is in purple and yellow for all the Arabidopsis genes and the novel genes respectively.

Three explanations that are not fully exclusive may be given to the rarely observed transcription of several novel genes. First, we may consider that some probes give an artefactual signal in one hybridization. Nevertheless, in any URGV-CATMA transcriptome experiment, a dye-swap is systematically done. A dye-swap is a technical repetition performed with the same biological samples and the two hybridizations of a dye-swap only differ by the dye, Cy3 or Cy5, tagging the two samples. Data for a probe are retained only if results are consistent in the two technical replicates. Furthermore, this explanation is also largely ruled out by the fact that we confirmed, by RT-PCR and sequencing, the transcription of 88% of the novel genes. Second, it is possible that the transcriptome approach allows, on rare occasions, the detection of an expression of genes constitutively expressed at low level. Indeed, constitutive genes always expressed at low level would generally give hybridization intensities below the thresholds for considering the corresponding probes as hybridized. It is only in a small number of experimental conditions when the expression is just slightly higher than in all other experimental conditions that the probes corresponding to these genes would be recognized as hybridized by the statistical method applied to the normalized data. Third, the signal responsible for the hybridizations that are unique but consistent within technical replicates might depend on relatively rare physiological or environmental situations. We tried to evaluate the relative explanatory potential of the last two expectations by comparing the distributions of intensity signals for both the whole genome and the novel genes. We expected that genes expressed constitutively but at low level would present a maximum signal hybridization intensity lower than the genomic distribution for this parameter. It is particularly clear that novel genes show the same relationship between the number of hybridized samples and the maximum signal intensity as the whole genome does (Figure 7). There is no novel gene for which the maximum intensity signal distinctly departs from the known genes showing the same number of hybridized samples. Thus, all together the transcriptome data for the novel genes suggest that the transcription of several of these genes are not only organ specific but also more specific to rare endogenous or environmental conditions than the whole genome. This double control of transcription might well explain our observation of transcription of several novel genes in only one biological sample. For this reason, the transcripts corresponding to these genes are less often present in the cDNA libraries which, in Arabidopsis, cover several organs but relatively few different environmental conditions.

### Erroneous gene merging

**Figure 8:**
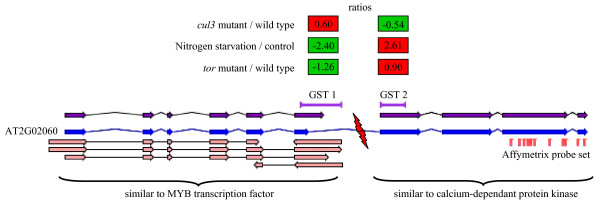
**Erroneous gene merging occurred in the annotation process and detected using CATMA transcriptome data**. The gene AT2G02060 is the fusion of two genes encoding a MYB transcription factor (gene 1) and a calcium-dependent protein kinase (gene 2). The opposite ratios concerning the two GSTs in 3 different transcriptome experiments (see CATdb database [16] for more information) highlight the erroneous merging. TAIR and Eugene CDS are represented by blue and purple arrows respectively. Cognate EST and cDNA supporting the MYB gene are represented by pink arrows.

In 422 loci, distinct GSTs match a single gene (not supported by full-length cDNA) according to the TAIR annotation but two different gene models were predicted by Eugene. For 13 loci, the transcriptome results show that two GSTs associated with the same gene provide opposite ratios in the same experiment, thus suggesting that they actually match two different genes (see Additional File 3). The fact that an erroneous gene merging has occurred during the automatic annotation process is reinforced by similarities with two distinct proteins. The example reported on Figure 8 shows that the TAIR predicted calcium-dependent protein kinase AT2G02060 (CDPK) corresponds to 2 Eugene predicted genes: a MYB motif containing gene and the CDPK, respectively corresponding to GST 1 and 2. In three independent experiments the two GSTs provide significant ratios (Bonferroni, P value<0.05) indicating that gene 1 is up-regulated while gene 2 is down-regulated in the same experiment. It also shows that the cognate Affymetrix probe set (from ATH1 chip) only reports the expression of the CDPK.

## Conclusion

The CATMA microarrays, based on both Eugene v1.0 and TIGR annotations, allowed us to discover 465 novel genes and to improve 29 gene models (16 CDS extensions and 13 gene splits). Furthermore, the analysis of the transcriptome data from 522 hybridized samples brings an additional functional dimension with numerous expression conditions of these novel and corrected genes. The biological and biochemical roles of the large majority of the novel genes remain unknown since only 15% of them share similarities with proteins of known function (Figure 3). However, the analysis of the large transcriptome data available through CATdb [16] may provide the first insights as to their functions. Inference on functions for unknown genes by such a compendium approach has already been successfully reported on yeast [23].

The fact that Eugene Markov model detects a high coding potential at these loci suggests that the novel genes are encoding proteins, with a short mean size (Figure 4), and are not RNA genes or huge extensions of neighbor gene UTRs. Despite recent works based on different methods [20,24], our results show that the "intergenic" section of the Arabidopsis genome is again reduced by the discovery of short genes characterized by a limited number of conditions promoting their expression. A recent application of the Affymetrix tiling array has recently highlighted novel transcribed regions in the Arabidopsis genome [24]. The intersection with our results concerns only 16 genes. The fact that the tiling approach missed several novel genes detected by our CATMA based approach might be explained by the comparatively limited number of mRNA samples used by Hanada et al. [24]. In April 2007, TAIR released the 7^th ^version of the annotation genome with 681 new genes compared to the previous one [25]. Only 70 genes out of the 465 novel genes identified by this work have been re-annotated at the structural level. As expected, these 70 genes are mainly those supported by cognate transcript sequences (see Additional File 1). All these results strongly illustrate that the annotation process is a long and difficult task and that many years are necessary after the first release of the sequence of a complex eukaryote genome to obtain (nearly) full knowledge of its gene content. Even 7 years after the publication of the complete sequence of the 5 Arabidopsis chromosomes [1], this goal has not yet been achieved. As our work shows, further progress requires the association of several and complementary approaches based on high-throughput experimental work and *ab initio *predictions. Due to the diversity of the possible approaches, in terms of confidence level and information content, the integration of the results is a process of increasing complexity that benefits a large community through the step-by-step updates of the Arabidopsis gene annotation previously done by TIGR [26] and pursued by TAIR [25].

## Methods

### Transcriptome data

The transcriptome data used in this work have all been produced with the CATMA v2 microarray [11]. They include 522 hybridized samples extracted from 40 different projects which cover 12 organ types: cells (61 samples), protoplasts (18), roots (78), hypocotyls (28), stems (10), leaves (136), flowers (10), mature pollen (2), siliques (4), seeds (16), aerial (40) or whole plants (119). Hybridizations include 49 specific developmental conditions, *i.e*. specific developmental stages and organs, 39 mutants and 63 different abiotic/biotic stresses or treatments. All the transcriptome data are available in the CATdb database [16]. They have also been deposited either in the NCBI GEO [27] or the EBI ArrayExpress [28] repositories (see additional file 4).

### Data normalization

For each CATMA array, the raw data are the logarithm of median feature pixel intensity at 635 nm (red) and 532 nm (green) wavelengths; no background is subtracted. A normalization per array is performed to remove systematic biases. First, spots that are considered badly formed features are excluded. Then, a global intensity-dependent normalization is performed using the lowess procedure [29] to correct the dye bias. Finally, for each block, the log-ratio median calculated over the values for the entire block is subtracted from each individual log-ratio value to correct effects on each block (print-tip, washing and/or drying effects). At the end of the normalization step, a normalized log-ratio, *i.e*. an expression difference (in log base 2) between the two samples co-hybridized on the same array, is given for each spot. A normalized logarithm intensity for each sample is also calculated. This is done according to the within-array correction proposed by Yang and Thorne [30].

Since each comparison of two samples is performed in dye-swap, the log-ratio between the two co-hybridized samples is defined as the average of the normalized log-ratios of the two arrays of a dye-swap, and the intensity signal of a sample is defined as the average of the normalized logarithm intensities of the two arrays of a dye-swap.

### Determination of the hybridized GSTs

We have developed a new statistical procedure to determine the set of probes whose intensity signal is considered significant, since existing procedures are either an arbitrary threshold based on an estimation of a local background or require the knowledge of a population of non-hybridized probes [31]. Our procedure is divided into two steps. The first step consists in the estimation of the intensity distribution using mixture models. The use of mixture of distributions appears natural, as each component of the mixture can be interpreted in terms of clusters of probes whose signal intensities are similar. Two characteristics of the histograms under study are first that the signal is bounded, the lower bound being linked to the auto-fluorescence of probes and second that an important number of probes have a signal close to the lower bound. This leads to dissymmetrical histograms with a left peak. For this reason, we use a truncated Gaussian mixture model in order to indirectly model the peak. The introduction of truncation parameters allows us to re-weight the densities on a compact support the bounds of which are defined by the minimal and maximal values of the intensity signal. The model parameters are estimated with a modified EM algorithm. To be specific, we modified the traditional EM algorithm proposed by Dempster [32] by including a fixed-point algorithm in the M-step to take into account the bias in the empirical estimators [33]. To best fit the histogram, a collection of mixture models of untruncated, left, right and left-right truncated Gaussian distributions is considered and, for each of them, the number of components varies between 1 and 5. The best model is chosen using the Bayes Information Criterion (BIC) [34]. The second step of our procedure is to define a hybridization threshold from the estimated density based on the components of the mixture. It is done as follows: when intensity values are ranked by descending order, the hybridization threshold is the first intensity value such that the Maximum *a posteriori *(MAP) rule does not classify it on the component with the highest mean and such that one of the calculated posterior probabilities is greater than 10^-4^. Once the threshold is defined, an intensity signal is declared as significant when it is greater than the hybridization threshold and the associated GST is declared hybridized. Otherwise, the intensity signal is not significant and we consider that transcription of the corresponding gene is not detected.

### Differential analysis for the detection of erroneous gene merging

We focus on distinct GSTs supposed to match the same gene, declared differentially expressed and which have log-ratio of opposite sign. To do that, a differential analysis is performed per dye-swap with a paired t-test on the normalized log-ratios. The number of observations per spot is inadequate for calculating a gene-specific variance. For this reason, it is assumed that the variance of the log-ratios is the same for all genes, and spots displaying extreme specific variances (too small or too large) are excluded. The raw P values are adjusted by the Bonferroni method, which controls the Family Wise Error Rate (FWER) [35]. When the Bonferroni P-value is lower than 0.05, the gene is declared differentially expressed. Genes with a missing P-value are genes with a too small or a too large specific variance or genes for which only one observation is available, *i.e*. when for one of the two arrays the spot corresponding to the gene was a badly formed feature.

### Data-mining

Searches of cognate transcripts, RACE-PCR products, previous lost annotation, and of homologous proteins in Arabidopsis or in other species have been carried out by sequence comparisons (BLASTn or BLASTp) with GenBank Release 159. Additional information such as PFAM motifs, MPSS tags, GST position and Eugene v1.59 CDS models have been retrieved from the FLAGdb^++ ^database [17,18].

### RT-PCR and sequencing

Primers for RT-PCR were designed using Primer3 [36] with the following parameters: primer size 20–21 mers, primer minimum Tm 50°C, primer maximum Tm 65°C, maximum Tm difference 3, primer minimum GC 40%, product minimum size 130, product optimal size 150, product maximum size 200. All other parameters were left at default values. The resulting primer sets are available in supplementary data (see Additional Files 1 and 2). Reverse transcription was performed on 2 μg of total RNA using an oligodT primer (18 mers) and the Superscript II reverse transcriptase (Invitrogen), for 1 hour at 42°C. The enzyme was then heat-inactivated at 65°C and the samples were treated with RNase H. Negative controls were performed without reverse transcriptase (RT-) on each sample with at least twenty couples of primers in order to check for any remaining DNA contamination. PCR amplifications were carried out from 2 μl of the RT product in the presence of 1 u of Taq DNA Polymerase (Biolab) in a 50 μl final volume, using the following program: hold for 5 min at 94°C; 35 cycles of 30 sec at 94°C, 30 sec at 58°C, and 30 sec at 72°C; and 7 min at 72°C; then 4°C. Ten μl of the RT-PCR products were run on a 2% agarose gel. The remaining part of the RT-PCR products was used for sequencing.

## Authors' contributions

SA designed the study, performed data-mining and drafted the manuscript. MLMM performed the statistical analyses. VB selected candidate GSTs and participated in exploitation of transcriptome data. LT carried out RT-PCR, participated in production of transcriptome data. FB and SB participated in production of transcriptome data. PEJ and MI provided RNA pollen samples. VT designed the GSTs and the primers for RT-PCR. TS developed the Eugene software and improved the manuscript. AL participated in design study and helped to draft the manuscript. JPR conceived of the study and generated transcriptome data. All authors have read and approved the final manuscript.

## Supplementary Material

Additional file 1 Information about identification and function of the 465 novel genes. 1: ID of GSTs selected outside TAIR models and exhibiting a transcription signal (with a web link to the CATdb database [16] for additional information). 2: Validation of the expression by sequencing RT-PCR product. 3: ID of CDS models proposed by Eugene at this locus. 4: ID of the gene upstream from the new gene. 5: Correction made in the recent TAIR annotation release 7. 6: ID of the gene downstream from the new gene. 7: Presence of cognate transcripts (EST and/or cDNA from GenBank R.159). 8: Presence of cognate MPSS tags [19]. 9: Presence of cognate RACE-PCR products obtained by TIGR [20]. Accession number is mentioned. 10: Presence of PFAM motifs (ID are mentioned). 11: Presence of homolog(s) in Arabidopsis based on BLASTX (GenBank R. 159). 12: Presence of homolog(s) in other species based on BLASTX (GenBank R. 159). 13: Putative biochemical function inferred from homology with known proteins. 14: Presence of a previous annotation carried out by AGI members at the BAC level but lost in the TAIR genome annotation. 15: Number of positive hybridized mRNA samples out of the 522 transcriptomes analyzed. 16: Minimum signal intensity detected. 17: Median signal intensity detected. 18: Maximum signal intensity detected. 19: Sequence of the left primer used in RT-PCR to confirm the transcriptional activity. 20: Sequence of the right primer used in RT-PCR to confirm the transcriptional activity. 21 to 32: Presence of hybridization signal in the following organs: leaf, root, stem, aerial, cell culture, pollen, seed, whole plant, flower, protoplast, silique, hypocotyl. Click here for file

Additional file 2 Information about extended genes. 1: ID of GSTs localized in the gene extension (CDS) and exhibiting a transcriptional signal (with a web link to the CATdb database [16] for additional information). 2: Validation of the expression by sequencing RT-PCR product. 3: ID of CDS models proposed by Eugene. 4: Correction made in the recent TAIR annotation release 7. 5: ID of the TAIR gene models. 6: Side of the extension. 7: Number of putative additional exons in CDS based on Eugene prediction. 8: Presence of cognate transcripts (EST and/or cDNA from GenBank R.159). 9: Presence of cognate MPSS tags [19]. 10: Presence of cognate RACE-PCR products obtained by TIGR [20]. Accession number is mentioned. 11: Presence of PFAM motifs (ID are mentioned). 12: Presence of homolog(s) in Arabidopsis based on BLASTX (GenBank R. 159). 13: Presence of homolog(s) in other species based on BLASTX (GenBank R. 159). 14: Putative biochemical function inferred from homology with known proteins. 15: Presence of a previous extension annotation carried out by AGI members at the BAC level but lost in the TAIR genome annotation. 16: Number of positive hybridized mRNA samples out of the 522 transcriptomes analyzed. 17: Minimum signal intensity detected. 18: Median signal intensity detected. 19: Maximum signal intensity detected. 20: Sequence of the left primer used in RT-PCR to confirm the transcription of the extension. 21: Sequence of the right primer used in RT-PCR to confirm the transcription of the extension. 22 to 33: Presence of hybridization signal in the following organs: leaf, root, stem, aerial, cell culture, pollen, seed, whole plant, flower, protoplast, silique, hypocotyl. Click here for file

Additional file 3 Information about detected erroneous gene merging. 1 and 2: ID of the two GSTs used to detect the gene merging (with a web link to the CATdb database [16] for additional information). 3: ID of the erroneous TAIR gene models. 4: Correction made in the recent TAIR annotation release 7. 5: Number of opposite hybridized mRNA samples between the two GSTs. 6: Additional information validating the erroneous gene merging (EST, homologies, MPSS). 7: Function deduced from homology with gene 1 (cognate to the GST 1). 8: Function deduced from homology with gene 2 (cognate to the GST 2) Click here for file

Additional file 4 Identification in GEO [27] or ArrayExpress [28] repositories of the 40 transcriptome projects used and web links to their detailed descriptions in the CATdb database [16]. Click here for file
